# The influence of the combined impairments and apical mesh surgery on the biomechanical behavior of the pelvic floor system

**DOI:** 10.3389/fbioe.2023.1292407

**Published:** 2024-01-08

**Authors:** Xianglu Xue, Qiuyu Zheng, Zhenhua Gao, Jihong Shen, Tingqiang Yao

**Affiliations:** ^1^ Faculty of Mechanical and Electrical Engineering, Kunming University of Science and Technology, Kunming, China; ^2^ The First Department of Urology, The First Affiliated Hospital of Kunming Medical University, Kunming, China

**Keywords:** combined impairments, pelvic floor system, suspension surgical mesh, magnetic resonance imaging, biomechanical behavior

## Abstract

**Objective:** The prolapse mechanism of multifactorial impairment of the female pelvic floor system and the mechanics of the pelvic floor after apical suspension surgery are not yet understood, so we developed biomechanical models of the pelvic floor for the normal physiological state (0°) and 90° pathological state.

**Methods:** Under different types and levels of the impairments and uterosacral suspensions, the possible changes in the morphometric characteristics and the mechanical characteristics of suspension and support functions were simulated based on the biomechanical models of the pelvic floor.

**Results:** After the combined impairments, the descending displacement of the pelvic floor cervix and the stress and displacement of the perineal body reached maximum values. After surgical mesh implantation, the stresses of the normal pelvic floor were concentrated on the uterine fundus, cervix, and top of the bladder and the stresses of the 90° pathological state pelvic floor were concentrated on the uterine fundus, uterine body, cervix, middle of the posterior vaginal wall, and bottom of the perineal body.

**Conclusion:** After the combined impairments, the biomechanical support of the bladder and sacrococcyx in the anterior (0°) and 90° pathological state pelvic floor system is diminished, the anterior vaginal wall dislodges from the external vaginal opening, and the posterior vaginal wall forms “kneeling” profiles. The pelvic floor system may evolve with a tendency toward the cervical prolapse with anterior and posterior vaginal wall prolapse and eventually prolapse. After surgical mesh implantation, the cervical position can be better restored; however, the load of combined impairment of the pelvic floor is mainly borne by the surgical mesh suspension, the biomechanical support function of pelvic floor organs and sacrococcyx was not repaired by the physiological structure, and the results of uterosacral suspension alone may be poor.

## 1 Introduction

Pelvic organ prolapse (POP) is the prolapse of the bladder or uterus or rectum from the vagina. Reportedly, the prevalence of POP in middle-aged and older female patients is 25%–41% (ATM et al., 2019), affecting 41%–50% of women aged over 40 years (Abhyankar et al., 2019). The prolapse mechanism of multifactorial impairment of the female pelvic floor system and the mechanics of the pelvic floor after the apical suspension surgery are not yet understood. Since POP has complex pathophysiological mechanisms, it is important to elaborate on the causes and pathogenesis of pelvic floor diseases from a biomechanical perspective, simulating different levels of impairments and reconstruction models of the pelvic floor.

The classical theories of the pelvic floor of POP include the “Three-Level Theory” proposed by DeLancey (1992) and the “Holistic Theory” and “Three-Chamber Theory” proposed by Petros and Woodman (2008). Under the guidance of these theories, a variety of apical suspension surgeries have been established for POP that can achieve better results but with higher complication and recurrence rates (Claerhout et al., 2009; Petri and Ashok, 2011). For example, there are 29% recurrences of uterine prolapse in hysteropexy and 54% recurrences of uterine prolapse in hysterectomy, and the vaginal angle and position are associated with prolapse recurrence (Bowen et al., 2023). Fundamentally, these theories basically explore POP pathogenesis from the histomorphology of female pelvic floor anatomy and the pathophysiology of POP, thus ignoring the mutual support function between soft tissue organs of the pelvic floor system and the biomechanical relationship between ligamentous fascial suspension support and the principle of the overall biomechanical equilibrium mechanism. The multi-structural impairment of the pelvic floor during the pathological process leads to the destruction of the pelvic floor mechanical equilibrium state, which may cause POP. Therefore, to overcome the treatment dilemma of POP, biomechanical methods are used to study the morphological features of female pelvic floor tissues and organs and the biomechanical mechanisms of pelvic floor mechanical support. This helps understand the overall mechanical equilibrium principle, mechanical imbalance principle, and repair and reconstruction principle of the female pelvic floor system in different states such as the normal physiological state, the combined impairment pathological state, and the postoperative repair and reconstruction state. To this end, we explore the internal biomechanical relationship between POP etiology and pathological results and provide biomechanical suggestion for the selection and improvement of the POP surgical plan.

Finite element analysis (FEA) is a computer technique used to solve engineering mechanics, physics, and mathematical problems that is commonly used to study the movement of tissues and organs in pathophysiological situations (Mayeur et al., 2016). Research on POP using FEM enables objective quantification and analysis of the tissue structure of various pelvic floor organs and support systems (Chen et al., 2009). By simulating different surgical approaches of the pelvic floor before and after pelvic floor surgical treatment, the results of stress and strain of organs and mechanical support structures are investigated. The clinical guidance on individualized surgical approaches and artificial implant materials can be provided (Knight et al., 2018; Silva et al., 2022). At present, there are few overall biomechanical models for the pelvic floor support system. FEA of POP has focused on one to a few support or suspension structures, such as the uterus, bladder, rectum, vagina, muscles, and ligaments (DeLancey, 2005; Petros and Woodman, 2008), without considering the overall support structures of the pelvic floor. However, the effects of multifactorial impairments of pelvic floor organs on the causes and pathogenesis of POP are still little discussed. Furthermore, the different levels of impairment and surgical reconstruction on the biomechanical characteristics of the pelvic floor system under multifactorial impairments are not yet understood. We have conducted related studies previously: (1) POP may be associated with mechanical imbalance and loss of physiological structural features of the pelvic floor by analyzing the rules of physiological angles from MR images (Li et al., 2022a; Wang et al., 2022); (2) the occurrence of POP may also be closely associated with the impairment of the supporting structures and disruption of the biomechanical equilibrium of the pelvic floor, and the biomechanics of the normal physiological state pelvic floor in the presence of single-organ impairment are discussed (Xie et al., 2023); and (3) the biomechanical mechanism of impairment and the pathogenesis of POP are investigated during the gradual evolution from the normal physiological structural state to the prolapse pathological state of the pelvic floor (Xue et al., 2023). However, we have not investigated the multifactorial impairment of the pelvic floor and the biomechanical mechanisms of clinical repair and reconstruction surgery. The displacements and the overall mechanical equilibrium principle of the normal pelvic floor system are determined by the combined actions of the physiological structural characteristics and the biomechanical characteristics. The biomechanical axis is the ideal axis that can maintain the overall biomechanical balance of the pelvic floor system and has the physiological angular characteristics of the uterus–cervix–vagina. The support function, i.e., the abdominal pressure and self-weight loads on the pelvic organs, is transmitted and transferred to the structural components of the support system through the combined biomechanical action of organ support and ligamentous suspension. The disruption of the mechanical balance of the female pelvic floor usually occurs during pregnancy and transvaginal delivery, causing irreversible pathological impairments to the female birth canal and pelvic floor support structures (Gautier et al., 2018). Furthermore, as women age, they inevitably experience loss of pelvic floor collagen, atrophy of the pelvic floor muscles, loss of muscle strength, and a decrease in vaginal elasticity and closing ability, leading to the disruption of the supporting structural components, which may ultimately disrupt normal pelvic floor mechanical homeostasis (Memon and Handa, 2012; Li et al., 2022b). All of this may be related to changes in the biomechanical axis and pelvic floor support function in the pelvic floor system, which may cause disorders such as POP. Therefore, the purpose of our study was to construct the 90° pathological state pelvic floor model from the normal physiological state pelvic floor (0°) model. In order to further understand the causes and pathogenesis of POP in the female pelvic floor, we would analyze the possible changes in the biomechanical axis and support function caused by the impairment factors, levels of impairments, and suspension surgery in the normal physiological and 90° pathological states of the pelvic floor system under the conditions of no-impairment, apical ligament impairment (the cardinal and uterosacral ligaments), combined impairments (simultaneous impairment of the cardinal ligament, uterosacral ligament, levator ani, and anterior vaginal wall), and surgical mesh implantation. In this paper, we first performed simulation calculations for the normal anteverted physiological state and the 90° pathological state of the pelvic floor at different levels of impairments to the apical ligament. Second, we explored the possible prolapse of the pelvic floor system in the normal physiological state and 90° pathological state at different impairment levels of combined impairments. Finally, we performed separate uterosacral suspensions of the pelvic floor system at different levels of combined impairments and analyzed the stress distribution features and stress transfer in the pelvic floor system. The present study is a complete extension of the previous seminal work.

## 2 Materials and methods

### 2.1 Geometric model of the pelvic floor

The included volunteer was a 35-year-old healthy nulliparous woman, with no previous history of pelvic floor dysfunction and pelvic floor surgery. All the MR images were acquired using a 3.0T MRI scanner in the supine resting state. Images were acquired consecutively in axial, sagittal, and coronal positions with the field of view of 20 cm × 20 cm, layer thickness of 4 mm, and layer spacing of 1 mm.

The MR images of the median sagittal plane of the pelvic floor of normal female volunteers were taken and imported into Mimics 21.0 software. Key characteristic discrete points with internal and external contours were marked using Mimics 21.0 software for each pelvic floor organ tissue by the senior imaging and urology surgeons. Finally, a 2D geometric model of the normal female pelvic floor system was obtained by cubic B-spline curve approximate fitting using SolidWorks software. According to our team’s pre-statistics of the uterovaginal angle and with reference to the uterovaginal angle measured on MR images in the pathological state (Li et al., 2022a), we constructed a predictive model of the uterus in the 90° pathological state based on the normal physiological state model. The angle of 90° for the pathological state model is based on the statistical selection of the uterovaginal angle from our MR images of the case in the previous stage. We investigated the evolution of pelvic floor biomechanics before and after surgery in the pathological state models, considering the process of pelvic floor changes at the moment of abdominal pressure loading, in order to predict the possible combined prolapse of the uterine orifice and vaginal wall. Therefore, the 90° pathological state pelvic floor model is the pelvic floor state of the intermediate transition process from the normal pelvic floor to the prolapsed pelvic floor in the pathogenesis of POP, and it is a predictive configuration of the progressive evolutionary process of the pathology from the normal to the prolapsed pelvic floor, which may predict the evolutionary trend of the mechanical imbalance of multifactorial impairments of the pelvic floor.

### 2.2 Mechanical properties and intrinsic structure relationships

Since the bladder–urethra, uterovaginal, perineal body, rectum, and levator ani units can produce large deformations, hyperelastic nonlinear material properties were used for the pelvic floor organs in this study (Peña et al., 2010). Martins et al. (2006) explained that the most appropriate models are Ogden, Yeoh, and Martins. Since the pelvic soft tissues such as the muscles and ligaments are composed of fibers, the tissues in the pelvic floor system are defined in this study as isotropic (Brandão et al., 2015), the perineal body is defined as compressible, and the remaining deformable structures are defined as incompressible. The advantage of the Yeoh model is that the higher-order form can reflect the inverse S-shaped stress–strain curve, which can simulate the sharp increase in material stiffness characteristics during the later stage, and the material constant values for the bladder–urethra, uterus–vagina, perineal body, and rectum in the pelvis are shown in Table 1 (Chen et al., 2015).

**TABLE 1:** Three-parameter Yeoh model hyperelastic material constants for each tissue.

Material factor	Uterus–vagina	Perineal body	Rectum	Bladder–urethra
C_10_ (MPa)	0.175	0.175	0.088	0.071
C_20_ (MPa)	8.648	8.684	3.092	0.202
C_30_ (MPa)	8.632	8.632	2.871	0.048
K (MPa)	16.0	16.0	11.0	9.0

**FIGURE 1:**
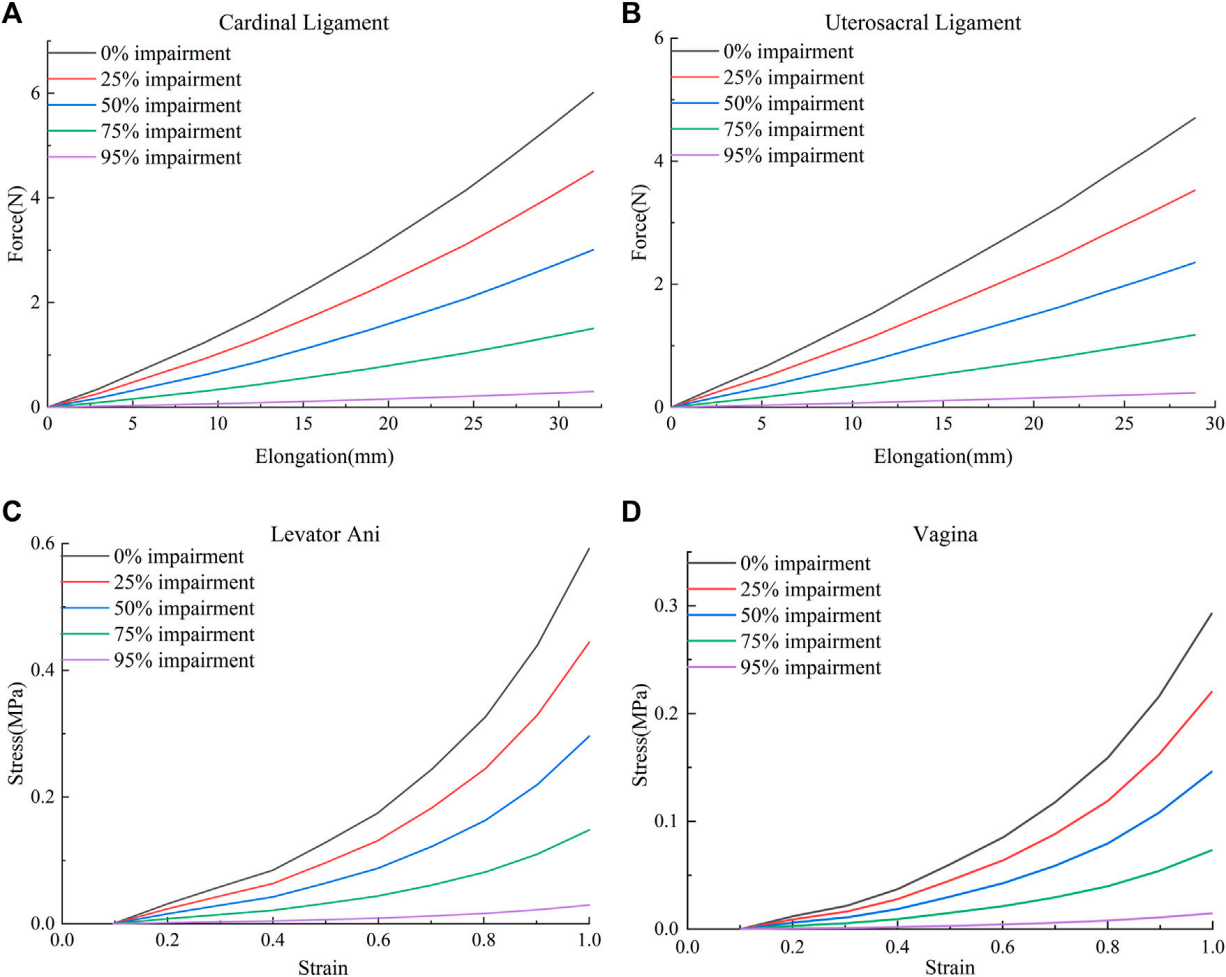
Mechanical curves of the cardinal ligament, uterosacral ligament, levator ani muscle, and anterior vaginal wall at different levels of impairments. **(A)** Force-elongation curves of the cardinal ligament; **(B)** Force-elongation curves of the uterosacral ligament; **(C)** Stress-strain curve of the levator ani muscle; **(D)** Stress-strain curve of the vagina.

The material parameters of levator ani muscle, cardinal ligaments, and uterosacral ligaments were derived from Luo et al. (2015). The Ogden (N = 2) hyperelastic constitutive model was used for the healthy levator ani muscle, and the material properties were μ_1_ = 0.0082 MPa, α_1_ = 0.1803, μ_1_ = 0.0216 MPa, and α_1_ = 15.112. Reducing the mechanical properties of the tissue materials allowed the simulation of combined impairments of the cardinal ligaments, uterosacral ligaments, levator ani muscles, and the anterior vaginal wall, and their impairments curves were obtained using the curve-fitting algorithm of Ansys software (25%, 50%, 75%, and 95%, respectively). Figure 1 shows the mechanical curves of the cardinal ligament, uterosacral ligament, levator ani, and anterior vaginal wall at different levels of impairments.

### 2.3 Two-dimensional finite element model of the female pelvic floor support system

#### 2.3.1 Finite element model construction

**FIGURE 2:**
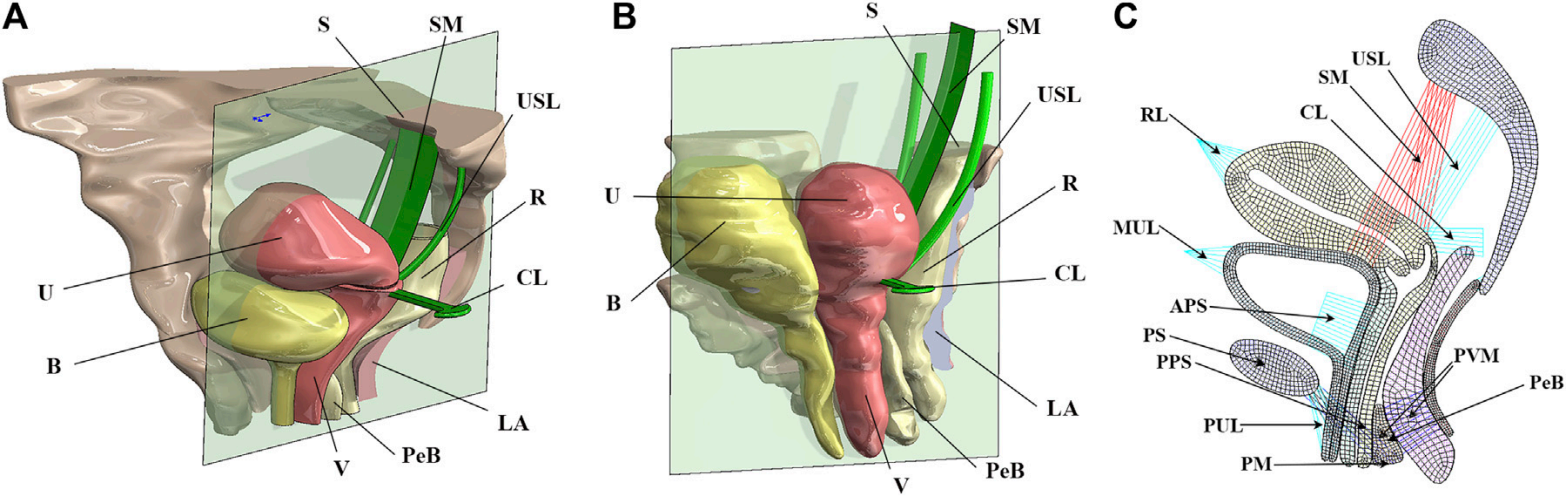
Schematic diagrams of the pelvic floor in the normal physiological and 90° pathological states and 2D equivalent finite element model of the normal pelvic floor. B, bladder; U, uterus; S, sacrum; SM, surgical mesh; USL, uterosacral ligament; R, rectum; CL, cardinal ligament; LA, levator ani; PeB, perineal body; V, vagina; RL, round ligament; MUL, median umbilical ligament; APS, anterior paravaginal support; PS, pubic symphysis; PPS, posterior paravaginal support; PUL, pubic urethral ligament; PM, perineal membrane; PVM, pubovisceral muscle. **(A)** Normal physiological state pelvic floor system after surgical suspension; **(B)** 90° pathological state pelvic floor system after surgical suspension; **(C)** 2D finite element model of the pelvic floor in normal physiological state.

We constructed a 2D model of the normal physiological state pelvic floor (Xue et al., 2023) based on MR images and computer-aided design methods. In this manner, we constructed a finite element model of the 90° pathological state pelvic floor. Figures 2A, B show the schematic diagrams of the normal physiological state and 90° pathological state pelvic floor with surgical suspensions, and Figure 2C shows the 2D finite element model of the pelvic floor and its equivalent ligamentous fascia in the normal physiological state of the anteverted position of the uterus.

SOLID185 elements were used to mesh the tissues and organs. Combination14 linear spring damping units were used to simulate the pull-supporting effect of the pelvic floor muscle groups and the suspension of the surgical mesh. The Combination39 nonlinear spring damping unit was used to simulate the cardinal ligaments and uterosacral ligaments. The surface contact method was used to simulate the mutual support of the organs in the pelvic floor. The stiffness parameters of the Combination14 linear spring damping unit for simulating the pelvic floor muscle groups were 0.1 N/m for the spring constant and 0.01 N·s/m for the damping coefficient. The parameters of the Combination39 nonlinear spring damping unit were obtained from the force–displacement curves of the cardinal and uterosacral ligaments shown in Figures 1A, B. In this paper, sacral suspension is simulated, and we focus on the effect of sacral suspension surgery on biomechanical repair of the pelvic floor. After communication between our team and the urology team of The First Affiliated Hospital of Kunming Medical University, most of the D1 position suspension is used in clinical practice and the effect is better, and the rest of the suspension position will have a bad surgical effect or difficult surgical operation and other problems. For example, D4 and D5 suspension positions have small operation spaces during clinical implementation, which is relatively difficult. Therefore, the D1 position suspension was selected for this study. The material properties of the surgical mesh in this paper were obtained from the inspection report of the China National Institutes for Food and Drug Control, and the specific parameters were as follows: the strip-type mesh could withstand a tensile force of 1.6 N/cm in both the transverse and longitudinal directions.

#### 2.3.2 Constraints, contact definitions, and loads

According to our previous study, we constrained the *Z*-direction displacement (perpendicular to the median sagittal plane) of all pelvic floor organ tissues, fixedly constrained the pubic bone and sacrococcyx, constrained the Y-direction displacement (vertical) of the pelvic floor bottom, and obtained the 90° pathological pelvic floor system by applying load in the X-direction (horizontal) at the top of the uterus in the normal pelvic floor system (Xue et al., 2023). According to Qiu (2017), we applied force loads in the X-direction (horizontal) at the bottom of the posterior vaginal wall to simulate the genital hiatus. The self-contact mechanics method was used to calculate the contact between the bladder and uterine vagina, anterior and posterior vaginal wall, posterior vaginal wall and rectum, rectum and levator ani, as well as the contact interaction between the internal surfaces of each tissue and organ. The standard frictionless, limited-slip, penalty-contact algorithm was used to determine and prevent penetration and large deformations between organs.

The aim of our simulations was to predict the deformation and the support of the pelvic floor organ structures in the normal physiological and 90° pathological states under different types and levels of impairments and surgical mesh implantation. For the applied intra-abdominal pressure, referring to Yang et al. (2016) and Xie et al. (2023), this study used the abdominal pressure of 8.2246 KPa during the supine Valsalva maneuver (83.9 cmH_2_O) as the loading load. Referring to the region of application of abdominal pressure in Ma et al. (2016), Yang et al. (2016), and Silva et al. (2021), we set the resultant force direction of abdominal pressure as the load direction, which is oblique from the upper part of the anterior abdominal wall toward the sacrococcyx. The areas of abdominal pressure loading for the pelvic floor system in the normal physiological and 90° pathological states were the uterus, bladder anterior wall, and upper part of the vaginal anterior wall.

## 3 Results

**FIGURE 3:**
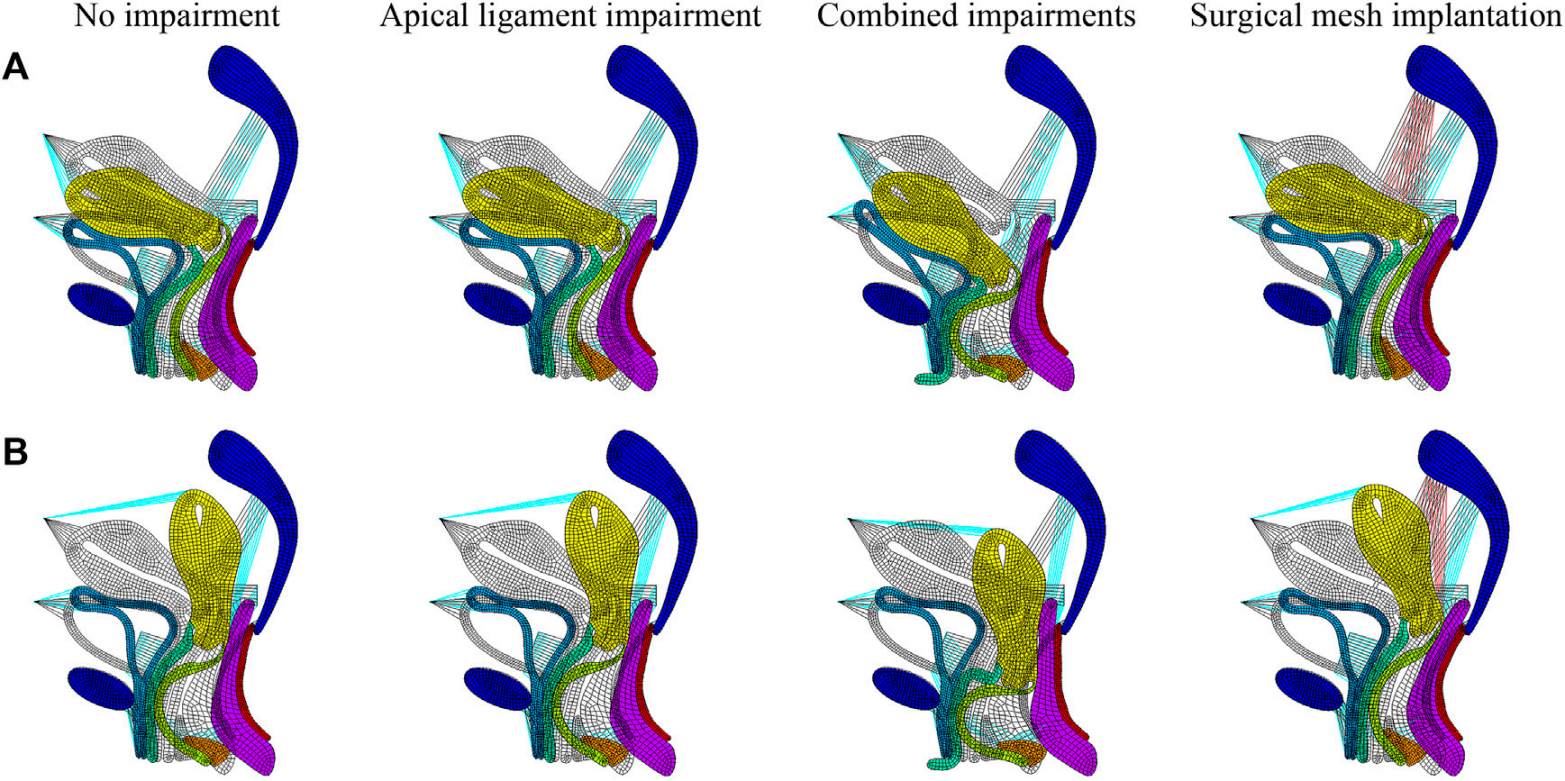
Morphology comparison of each tissue before and after deformation in the normal and 90° pathological pelvic floor systems under the conditions of no-impairment, 50% apical ligament impairment, 50% combined impairment, and surgical mesh implantation. **(A)** Normal physiological state pelvic floor system (0°); **(B)** 90° pathological state pelvic floor system.

We performed numerical simulations of finite element models of the pelvic floor system in the normal physiological and 90° pathological states under the conditions of no-impairment, apical ligament impairment, combined impairments, and surgical mesh implantation. Figure 3 shows the comparison before and after deformation of each tissue in the normal physiological and 90° pathological states of the pelvic floor system in no-impairment, 50% apical ligament impairment, 50% combined impairment, and surgical mesh implantation under the supine Valsalva maneuver (83.9 cmH_2_O) with abdominal pressure. The black shaded area represents the initial position, and the colored area represents the position after deformation. Figure 3A shows the normal physiological state pelvic floor system (0°), and Figure 3B shows the 90° pathological state pelvic floor system.

The transition from red to blue in the displacement and stress figures represents a gradual decrease in displacement or stress. The stress in the stress figures is identified as “von Mises stress,” and the displacement in the displacement figures is “displacement vector sum.” We simulated that under combined impairments of the cardinal and uterosacral ligaments, levator ani, and anterior vaginal wall, the biomechanical support of the bladder is diminished and the middle segment of the posterior vaginal wall is flexed and deformed toward the pubic bone to form “kneeling” profiles with a greater physiological angle. This causes the uterus to move from its natural position and press against the vaginal wall, and the normal physiological and 90° pathological states of the pelvic floor system may tend toward cervical prolapse with anterior and posterior vaginal wall prolapse. The 90° pathological state pelvic floor is compared to the normal pelvic floor, where the physiological angle of the uterus–vagina is lost and the uterine orifice is oriented in the vertical downward direction toward the vaginal opening, which may exacerbate the soft tissue impairment and biomechanical imbalance of the pelvic floor and result in greater prolapse.

**FIGURE 4:**
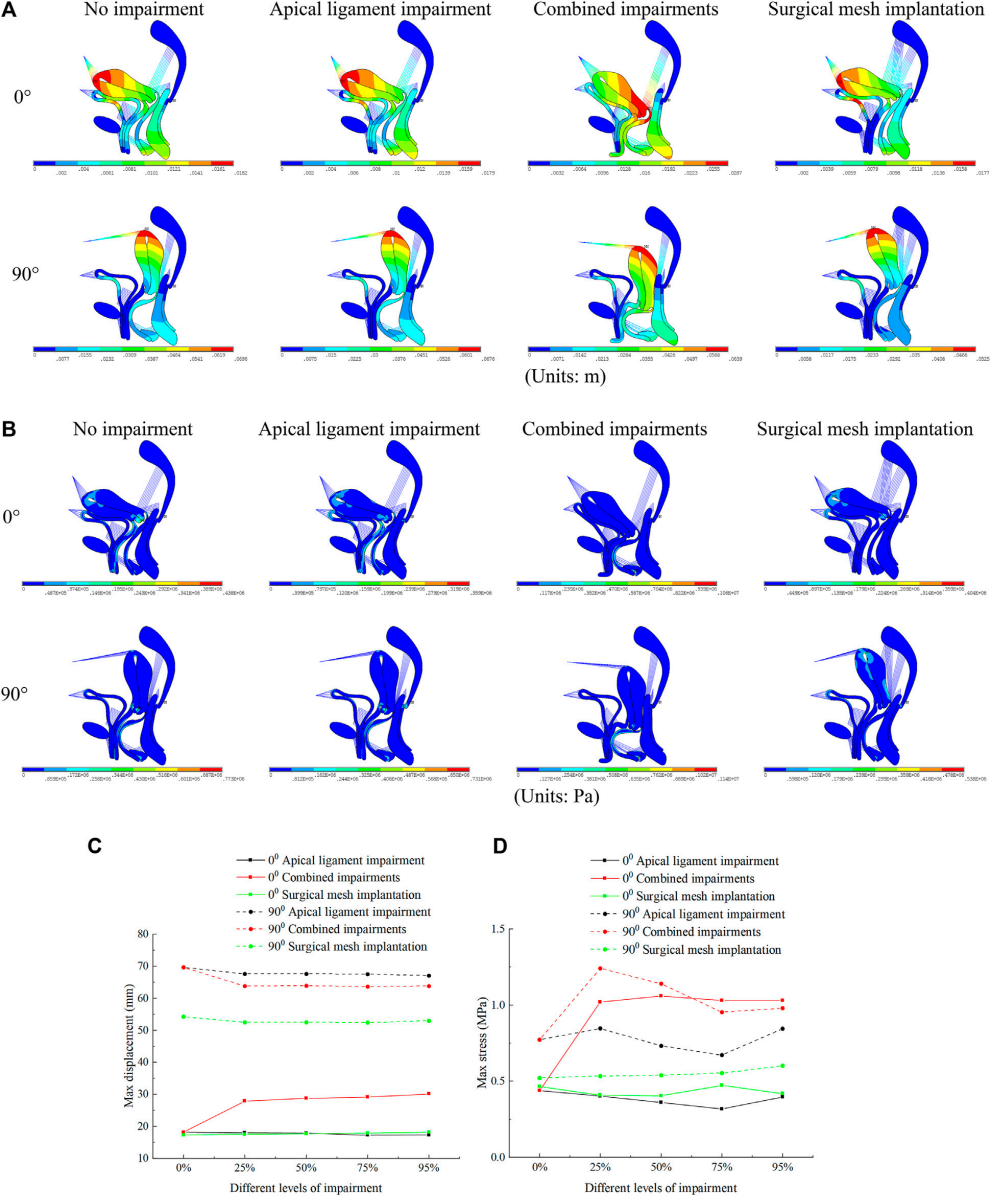
Total displacement figure, total stress figure, total displacement, and total stress change patterns of the normal and 90° pathological pelvic floor systems under the conditions of no-impairment, 50% apical ligament impairment, 50% combined impairment, and surgical mesh implantation. **(A)** Total displacement figure of the pelvic floor system; **(B)** Total stress figure of the pelvic floor system; **(C)** Patterns of change of total displacement of pelvic floor system; **(D)** Patterns of change of total stress of pelvic floor system.

Our simulation results show that the bladder plays an important supporting role in the normal pelvic floor system, and the bladder plays no significant supporting role for the uterus in the 90° pathological pelvic floor system. The displacement regions are mainly distributed at the uterus, bladder, vagina, perineal body, rectum, and levator ani, as shown in Figure 4A. The stress regions are mainly distributed at the cervix, middle and upper segments of the anterior vaginal wall, middle segment of the posterior vaginal wall, and perineal body, as shown in Figure 4B. The total displacement of the 90° pathological pelvic floor system was greater than that of the normal pelvic floor system (0°), and there was some reduction in total displacement and total stress after surgical mesh implantation relative to the impairment condition. The total displacement and total stress were greatest in the normal pelvic floor system with combined impairments, and there was some reduction after surgical mesh implantation, as shown in Figures 4C, D.

**FIGURE 5:**
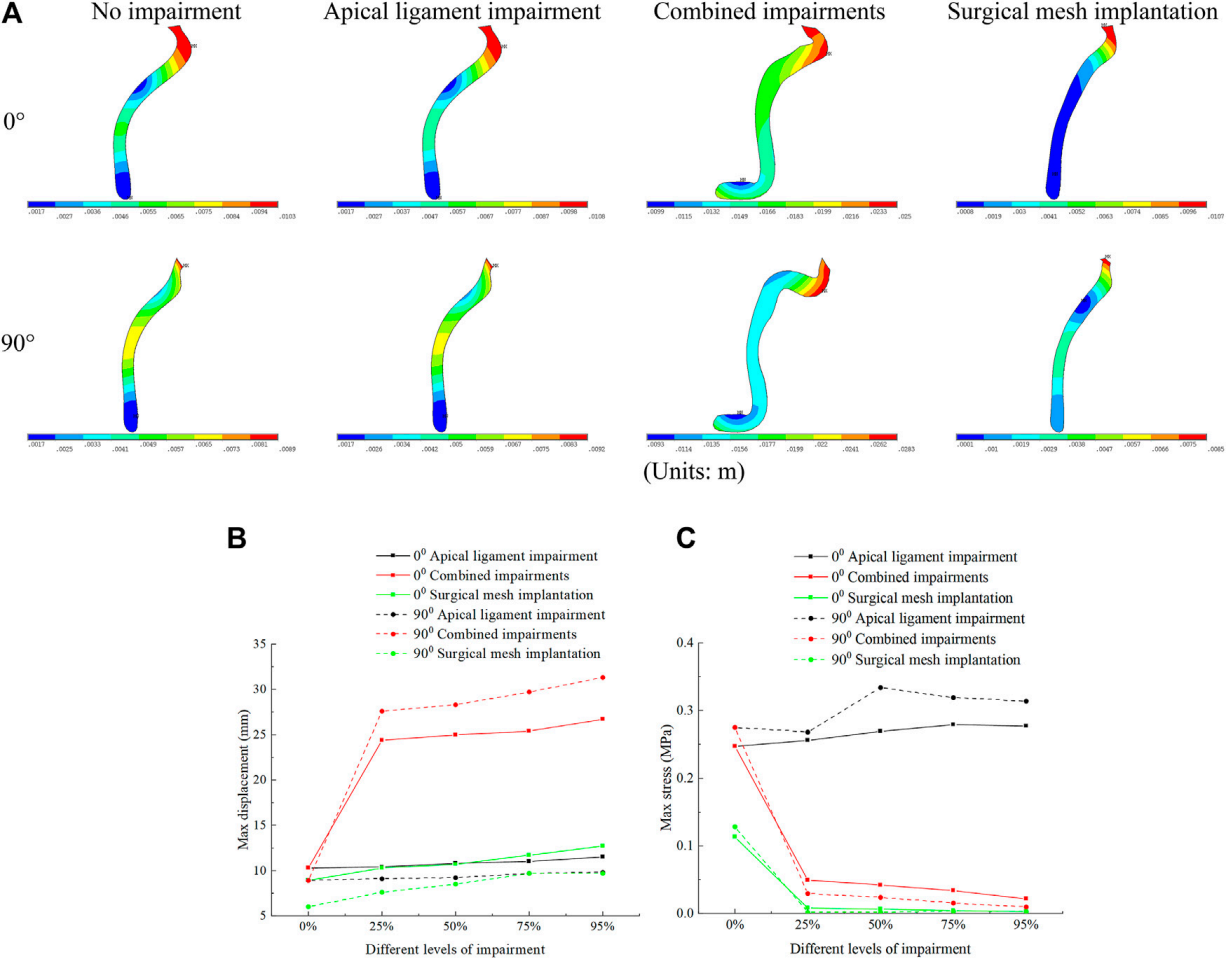
Displacement figure and displacement and stress change patterns of the anterior vaginal wall in the normal and 90° pathological pelvic floor systems under the conditions of no-impairment, 50% apical ligament impairment, 50% combined impairment, and surgical mesh implantation. **(A)** Displacement figure of the anterior vaginal wall; **(B)** Patterns of change of displacement of the anterior vaginal wall; **(C)** Patterns of change of stress of the anterior vaginal wall.

In the combined impairments, the displacement of the anterior vaginal wall of the normal and 90° pathological state pelvic floor systems was greatest, which continued to increase with the levels of impairment. At this time, the anterior vaginal wall prolapse occurred in our model. The difference in displacement of the anterior vaginal wall was not significant after apical ligament impairment and surgical mesh implantation. In combined impairments, the displacements of the anterior vaginal wall in the normal physiological state (0°) of the pelvic floor system at different levels of impairment were 10.3 mm, 24.4 mm, 25 mm, 25.4 mm, and 26.7 mm, respectively, and the displacements of the anterior vaginal wall in the 90° pathological state of the pelvic floor system at different levels of impairment were 8.9 mm, 27.6 mm, 28.3 mm, 29.7 mm, and 31.3 mm, respectively. We predict that the 90° pathological state pelvic floor system is more prone to prolapse. The stresses on the anterior vaginal wall in the normal and 90° pathological state pelvic floor systems reach a maximum value with apical ligament impairment. The stresses on the anterior vaginal wall in the event of combined impairments decrease dramatically, and the stress is then transferred to the supporting structures of the pelvic floor system. After surgical mesh implantation, the stresses on the anterior vaginal wall were transferred to the suspensory mesh without anterior wall prolapse. We consider this to be due to the anterior vaginal wall exerting some self-supporting action, but when impairment of the anterior vaginal wall occurs, the stresses on the anterior vaginal wall are transferred to the rest of the supporting organs, as shown in Figure 5.

**FIGURE 6:**
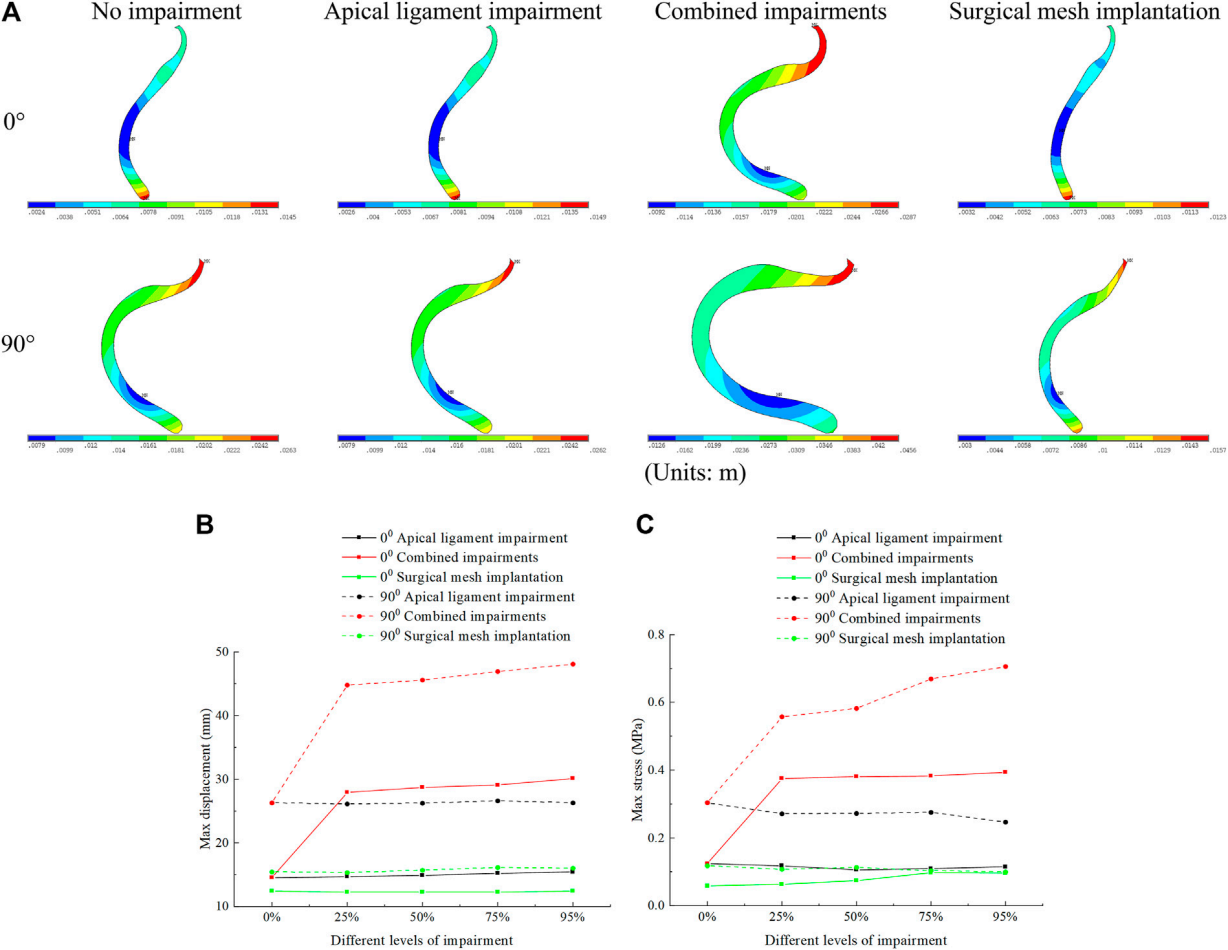
Displacement figure and displacement and stress change patterns of the posterior vaginal wall in the normal and 90° pathological pelvic floor systems under the conditions of no-impairment, 50% apical ligament impairment, 50% combined impairment, and surgical mesh implantation. **(A)** Displacement figure of the posterior vaginal wall; **(B)** Patterns of change of displacement of the posterior vaginal wall; **(C)** Patterns of change of stress of the posterior vaginal wall.

Displacement of the posterior vaginal wall occurred mainly at the superior and inferior segments, and stresses were mainly distributed at the middle segment. The displacement and stresses of the posterior vaginal wall in the normal physiological and 90° pathological states of the pelvic floor system in the combined impairments were greater than those in the apical ligament impairment, and the displacement and stresses of the posterior vaginal wall reached a minimum value after surgical mesh implantation. In combined impairments, the posterior vaginal wall stresses in the normal physiological (0°) state pelvic floor system at different levels of impairment were 0.124 MPa, 0.375 MPa, 0.381 MPa, 0.383 MPa, and 0.394 MPa, respectively, and the posterior vaginal wall stresses in the 90° pathological state pelvic floor system at different levels of impairment were 0.304 MPa, 0.558 MPa, 0.582 MPa, 0.669 MPa, and 0.706 MPa, respectively. The posterior vaginal wall stress after combined impairments is greater than that in no-impairment. Likewise, we consider that the posterior vaginal wall exerts self-supporting action, and the support of the anterior vaginal wall decreases after the impairment and the support of the posterior vaginal wall increases, leading to increased stress. Therefore, the displacement and stress of the posterior vaginal wall in combined impairments are greater than those in apical ligament impairment and no-impairment. The displacement and stress of the posterior vaginal wall in the 90° pathological state pelvic floor system are greater than those in the normal pelvic floor system. We believe that this is because the physiological angle of the uterus is altered and the biomechanical support of the bladder is weakened in the 90° pathological state pelvic floor system, and some of the stress is transferred to the posterior vaginal wall, resulting in increased stress on the posterior vaginal wall, as shown in Figure 6.

**FIGURE 7:**
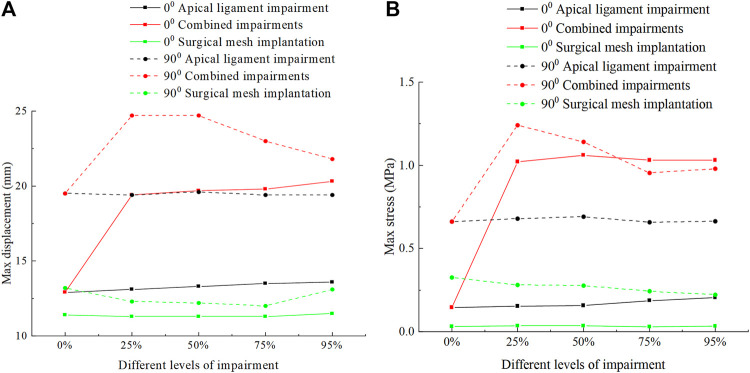
Change patterns of the perineal body displacement and stress in the normal and 90° pathological pelvic floor systems under the conditions of no-impairment, apical ligament impairment, combined impairments, and surgical mesh implantation. **(A)** Patterns of change of the perineal body displacement; **(B)** Patterns of change of the perineal body stress.

Figure 7 shows the displacement and stress change patterns of the perineal body in normal and 90° pathological state pelvic floor systems in no-impairment, apical ligament impairment, combined impairments, and surgical mesh implantation. The displacement and stresses of the perineal body were mainly distributed at the bottom of the perineal body. The displacement and stress of the perineal body in the normal and 90° pathological states of the pelvic floor system in combined impairments are greater than those in the apical ligament impairment, and the displacement and stress of the perineal body reach the minimum value after surgical mesh implantation. We consider that this is because the support of the vaginal wall is weakened after anterior vaginal wall impairment, and part of stress on the pelvic floor is transferred to the perineal body, resulting in increased stress in the perineal body compared to the apical ligament impairment. In the combined impairments, the perineal body stresses in the normal physiological state (0°) of the pelvic floor system at different levels of impairment were 0.146 MPa, 1.02 MPa, 1.06 MPa, 1.03 MPa, and 1.03 MPa, respectively, and the perineal body stresses in the 90° pathological state of the pelvic floor system at different levels of impairment were 0.662 MPa, 1.24 MPa, 1.14 MPa, 0.954 MPa, and 0.979 MPa, respectively. The stresses on the perineal body increased in the combined impairments compared to those in no-impairment. The stresses on the perineal body in the 90° pathological state pelvic floor system were basically greater than those in the normal physiological state pelvic floor system at different levels of impairment. We believe that this is because the physiological angle of the uterus is changed in the 90° pathological state pelvic floor system, the biomechanical support of the bladder is weakened, and part of the stress is transferred to the perineal body, resulting in increased stress on the perineal body. All of the above findings indicate that the perineal body plays an important supporting role in the pelvic floor support system.

**FIGURE 8:**
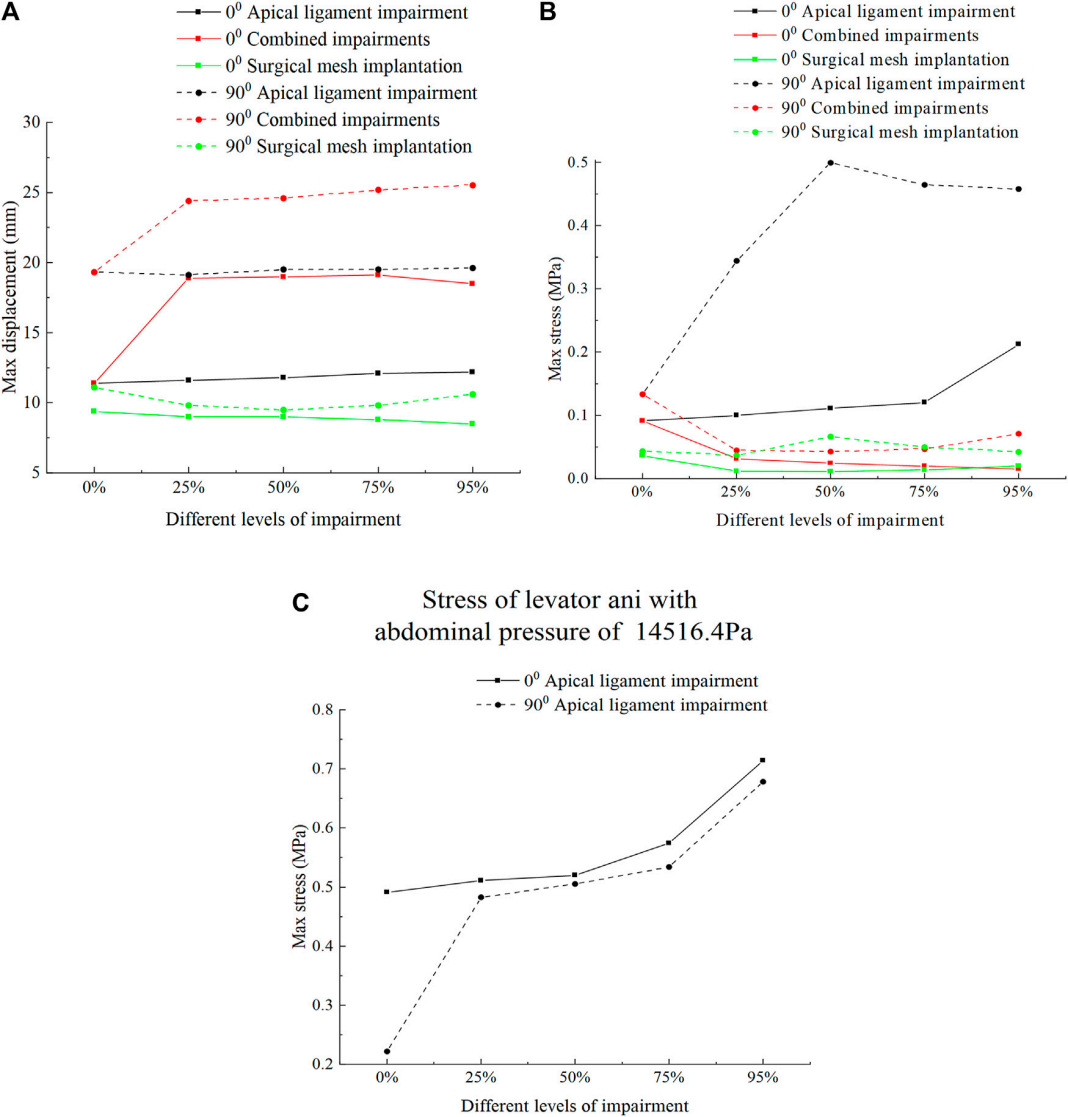
Change patterns of levator ani displacement and stress in the normal and 90° pathological pelvic floor systems under the conditions of no-impairment, apical ligament impairment, combined impairments, and surgical mesh implantation. **(A)** Patterns of change of the levator ani displacement; **(B)** Patterns of change of the levator ani stress; **(C)** Change patterns of levator ani stress in the normal and 90° pathological state pelvic floor systems under an abdominal pressure of 14516.4 Pa in the apical ligament impairment.

**FIGURE 9:**
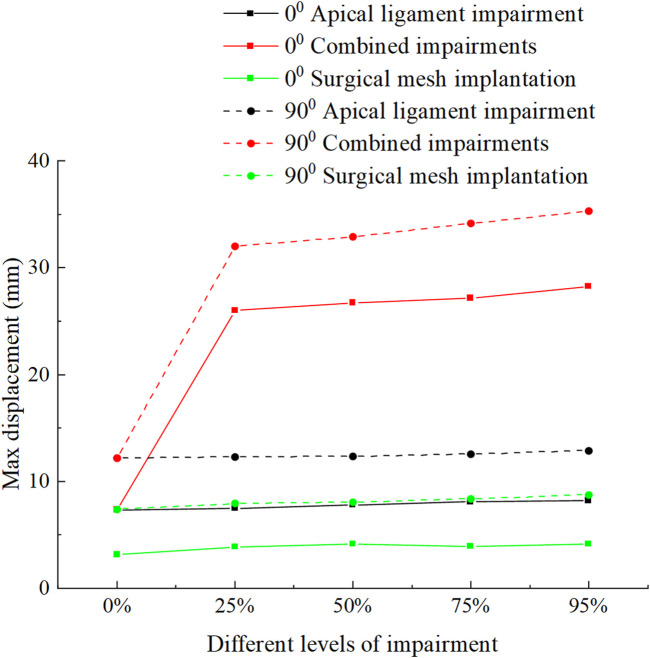
Change patterns of cervical descending displacement in the normal and 90° pathological pelvic floor systems under the conditions of no-impairment, apical ligament impairment, combined impairments, and surgical mesh implantation.

Figure 8 shows the displacement and stress change patterns of the levator ani muscle in the normal and 90° pathological state pelvic floor systems in no-impairment, apical ligament impairment, combined impairments, and surgical mesh implantation. The stresses of the levator ani muscle were mainly distributed in the upper segment of the levator ani muscle attached to the sacrum, while the displacements were mainly distributed in the bottom of the levator ani muscle. The displacement and stress of the levator ani muscle in the 90° pathological pelvic floor system are greater than those of the levator ani muscle in the normal pelvic floor system. We consider that the 90° pathological state of the pelvic floor is under 8.2246 KPa of abdominal pressure due to the weakening of bladder support, and part of the stress is shifted to the sacrococcyx and other supportive points, resulting in increased stress on the top of the levator ani. However, under abdominal pressure (Standing Valsalva: 14.5164 KPa) and greater abdominal pressure, the stress at the top of the levator ani of the normal physiological state pelvic floor is greater than that of the 90° pathological state pelvic floor, and the support of the sacrococcyx in the normal pelvic floor system is enhanced and greater than that in the 90° pathological state pelvic floor, as shown in Figure 8C. The 90° pathological state pelvic floor may prolapse under greater abdominal pressure due to inadequate support at the bottom of the sacrococcyx. The displacement of the levator ani in the normal and 90° pathological states of the pelvic floor system was greater in the combined impairments than that in the apical ligament impairment, while the stress of the levator ani in the apical ligament impairment was greater than that in the combined impairments. We consider that this is because the physiological position of the uterus in the pelvic floor system is changed after combined impairments, resulting in a change in the mechanical equilibrium relationship with a transfer of stresses from the levator ani to other supporting structures; thus, the stresses on the levator ani muscle are less during combined impairments. After surgical mesh implantation, the displacement of the levator ani reaches its minimum value and the stresses on the levator ani are transferred to the suspension mesh. In apical ligament impairment, the stresses of the levator ani in the normal physiological state (0°) of the pelvic floor system at different levels of impairment were 0.0916 MPa, 0.0996 MPa, 0.111 MPa, 0.12 MPa, and 0.212 MPa, respectively, and the stresses of the levator ani in the 90° pathological state of the pelvic floor system at different levels of impairment were 0.133 MPa, 0.344 MPa, 0.499 MPa, 0.464 MPa, and 0.458 MPa. The stress of the levator ani muscle was increased in the apical ligament impairment compared with that in no-impairment. The above findings indicate that the levator ani muscle plays an important supporting role in the pelvic floor support system. Figure 9 shows the change patterns of cervical descending displacement in normal and 90° pathological state pelvic floor systems under the conditions of no-impairment, apical ligament impairment, combined impairments, and surgical mesh implantation. The figure shows the normal and 90° pathological states of the pelvic floor system in combined impairments, with the cervical descending displacement increasing with the increase in the impairment levels, and the cervical descending displacement is the largest at this time. The increase in the impairment levels had no significant effect on the cervical descending displacement in apical ligament impairment and surgical mesh implantation, but the cervical descending displacement after surgical mesh implantation was smaller than that in apical ligament impairment. In combined impairments, the cervical descending displacements in the normal physiological state (0°) of the pelvic floor system at different levels of impairment were 7.299 mm, 25.99 mm, 26.723 mm, 27.146 mm, and 28.214 mm, respectively, and the cervical descending displacements in the 90° pathological state of the pelvic floor system at different levels of impairment were 12.18 mm, 31.985 mm, 32.892 mm, 34.124 mm, and 35.301 mm, respectively. When the surgical mesh was implanted, the cervical descending displacements of the normal pelvic floor system at different levels of impairment were 3.15 mm, 3.869 mm, 4.12 mm, 3.945 mm, and 4.107 mm, respectively, and the cervical descending displacements of the 90° pathological state pelvic floor system at different levels of impairment were 7.396 mm, 7.93 mm, 8.062 mm, 8.35 mm, and 8.75 mm, respectively. The above data indicate that the cervical descending displacement of the 90° pathological state pelvic floor system is greater than that of the normal pelvic floor system, and we predict that the 90° pathological state of the pelvic floor system is more prone to prolapse.

## 4 Discussion

The focus of this study is based on the previous two-dimensional equivalent finite element model of the pelvic floor. We construct the two-dimensional biomechanical model of the pelvic floor by considering the influences of the combined impairments and apical mesh surgery and investigate the biomechanical behavior of the pelvic floor system in the physiological state (normal anteverted forward flexion position) and 90° posterior rotation of the uterine–vaginal angle. Under the conditions of different impairment types, different impairment levels, and surgical mesh implantation, the stress distribution and morphological feature deformation patterns of the pelvic floor system are analyzed, and the possible changes in the biomechanical axis and support of the pelvic floor are discussed and thus the POP diseases that may be caused. The study in this paper is based on the two-dimensional biomechanical model with selected boundary conditions and material parameters. Under abdominal pressure loading, we simulated the changes in the normal and 90° pathological state pelvic floor systems under four conditions: no impairment, apical ligament impairment, combined impairments, and surgical mesh implantation of the combined impairment. We explored the internal biomechanical essence of the normal and impaired pelvic floor system in the current investigation. It can be concluded that the normal pelvic floor system achieves a state of biomechanical balance and self-locking morphological features through morphological changes in tissues and organs. Abdominal pressure and self-weight loading lead to a subsequent change in the uterine–cervical angle and the mid-lower segment angle of the vagina, which triggers the self-locking angle feature of the uterine–cervical–vaginal physiological structures and in turn maintains the morphological stability and biomechanical axis of the pelvic floor. However, in the case of multifactorial impairments of pelvic floor support, the original biomechanical balance state of the pelvic floor support system may be disrupted, resulting in mechanical imbalance and combined force direction changes in the pelvic floor. As a result, the changes in pelvic floor support, uterovaginal shape features, and the loss of pelvic floor self-locking morphological angle features occur, leading to pelvic floor dysfunctional diseases. Considering the internal biomechanical essence of the pelvic floor system, restoring the normal morphologic features of the uterovaginal axis may contribute to achieving the treatment effects for pelvic floor dysfunction disease (Ye-yuan, 2020).

In no-impairment and apical ligament impairment, the normal anteverted (0°) female pelvic floor is centered on the uterus–cervix–vagina, and the pubic bone and bladder provide anterior pelvic support. The cervix, levator ani, and sacrococcyx terminalis provide apical support, and the perineal body, rectum, and levator ani provide posterior pelvic support. The ligamentous fascia provides adjunctive suspension and coordinating function. The abdominal pressure and self-weight loading are borne by the combined action of the support and the suspension of the pelvic floor. After combined impairments, the biomechanical support of the bladder and sacrococcyx is weakened, the cervical descending displacement reaches its maximum value, and the stress and displacement of the perineal body reach their maximum values. This causes the uterus to move from its natural position and press on the vaginal wall. The anterior vaginal wall reaches maximum displacement and prolapses from the external vaginal opening, and the posterior vaginal wall forms “kneeling” profiles. The pelvic floor system may evolve with a tendency toward cervical prolapse with anterior and posterior vaginal wall prolapse, eventually leading to prolapse, which is in contrast to our previous study (Xue et al., 2023), and may lead to prolapse of the anterior vaginal wall due to increased anterior vaginal wall impairment.

The biomechanical axis of the 90° pathological state pelvic floor may gradually shift from the sacrococcygeal direction to the direction of the external vaginal opening. The uterine–cervical–vaginal axis is collinear and directed toward the external vaginal opening. The uterus overlaps the bladder support, and the pelvic floor is under great stress. However, the perineal body continues to provide support. After combined impairments in the 90° pathological state of the pelvic floor, the loss of uterovaginal physiological angle is more marked, with greater anterior vaginal wall displacement, posterior vaginal wall displacement and stress, perineal body displacement, and cervical descending displacement than those in a normal anteverted pelvic floor. This causes loss of the self-locking morphometric angle features of the pelvic floor system and biomechanical support of the bladder, progressively exacerbating pelvic floor soft tissue impairments and biomechanical imbalances. Thus, we predict that the 90° pathological state of the pelvic floor is more prone to prolapse than the normal anteverted pelvic floor, especially occurring with combined impairments and loss of biomechanical support functions, which is consistent with our previous study (Xue et al., 2023).

After surgical mesh implantation in the normal pelvic floor system, the cervical descending displacement is significantly reduced, and the regions of stress distribution are concentrated on the uterine fundus, cervix, and top of the bladder. Due to the suspension action of the surgical mesh on the cervix, the stress of the cervix increases, the stress of the perineal body decreases, and the support of the perineal body on the middle and lower segments of the vagina and the middle segment of the urethra is weakened. After uterosacral suspension in the 90° pathological state of the pelvic floor combined impairment model, the stresses are concentrated on the uterine fundus, uterine body, cervix, middle segment of the posterior vaginal wall, and bottom of the perineal body. Both the normal physiological state and the 90° pathological state of the pelvic floor system indicate that the mesh in apical suspension surgery mainly bears the abdominal pressure load of the pelvic floor and the self-weight load of the organ, and the action direction of the force is toward the vertical prolapse of the surgical mesh suspension–vaginal opening. Thus, once the abdominal pressure of the pelvic floor system or the combined impairment factor and levels are increased, the results of simple uterosacral suspension may be poor. The surgery only restores the anatomical position of the cervix better, but it also limits the mobility of the cervix, and does not provide a good repair and reconstruction effect to the biomechanical axis and support function of the pelvic floor system with relatively severe prolapse.

The 2D equivalent mechanical model of the female pelvic floor we constructed is the 2D projection model based on the median sagittal plane, which may be able to predict the prolapse tendency of the female pelvic floor and the biomechanical support changes in the surgical reconstruction. However, there are some limitations to our study. First, the finite element model we constructed is based on certain assumptions, including but not limited to boundary conditions. Second, the levator ani muscle structure in this study was simplified to the levator plate, and the mesh was simplified to the spring model, but it could represent the biomechanical behavior of the pelvic floor to some extent. Finally, we ignored the active contraction of the levator ani muscle (Brandão et al., 2016). Despite some limitations in our study, we fully considered the mechanical properties of the hyperelastic materials of the pelvic floor system to simulate the support function impairments of the pelvic floor and the interaction laws between organs and tissues after sacral suspension surgery. This can improve the accuracy of the study of the mechanical mechanism after pelvic floor support function impairment and surgical repair.

## 5 Conclusion

In summary, this paper reconstructs the two-dimensional biomechanical model of the female pelvic floor in the normal physiological and 90° pathological states with surgical mesh implantation based on MR images. In the normal pelvic floor system, the uterus–cervix–vagina plays an important role in self-locking physiological angle feature, which transfers the abdominal pressure and self-weight load to the combined force axis direction of the sacrococcygeal curvature. This conforms to the overall biomechanical balance of physiologic structures and forms the principle of pelvic floor self-locking. The 90° pathological state of the pelvic floor system shows the loss of the uterine–vaginal physiological angle, the change in the combined force axis direction to point toward the vaginal opening, and the loss of the self-locking morphological features, which may cause the mechanical homeostatic imbalance. In addition, the support of the support system structural components is diminished in both normal physiological and 90° pathological states of the pelvic floor after combined impairments, which may aggravate the occurrence of pelvic floor disorders. After surgical mesh implantation in the normal anteversion (0°) and 90° pathological states of the pelvic floor system, the cervical position was better restored. However, the load of the combined impairment pelvic floor model was mainly borne by the surgical mesh suspension, and the direction of force was oriented toward the vertical prolapse of the surgical mesh suspension–vaginal opening. The repair and reconstruction of the biomechanical axis and support function of the pelvic floor system may not be better improved by surgery, and the results of uterosacral suspension alone may be poor. Therefore, pelvic floor reconstruction surgery should aim to repair the normal biomechanical axis of the uterus–vagina and the mechanical support function, enabling the pelvic floor to achieve a normal physiological balance.

## Data Availability

The original contributions presented in the study are included in the article/Supplementary Material; further inquiries can be directed to the corresponding author.
